# LiCo_2_As_3_O_10_: une nouvelle structure à tunnels inter­connectés

**DOI:** 10.1107/S1600536813013548

**Published:** 2013-05-31

**Authors:** Youssef Ben Smida, Abderrahmen Guesmi, Ahmed Driss

**Affiliations:** a Laboratoire de Matériaux et Cristallochimie, Faculté des Sciences, El Manar II, 2092 Tunis, Tunisia; b Institut Préparatoire aux Etudes d’Ingénieurs d’El Manar, BP 244 El Manar II, 2092 Tunis, Tunisia

## Abstract

The title compound, lithium dicobalt(II) triarsenate, LiCo_2_As_3_O_10_, was synthesized by a solid-state reaction. The As atoms and four out of seven O atoms lie on special positions, all with site symmetry *m*. The Li atoms are disordered over two independent special (site symmetry -1) and general positions with occupancies of 0.54 (7) and 0.23 (4), respectively. The structure model is supported by bond-valence-sum (BVS) and charge-distribution (CHARDI) methods. The structure can be described as a three-dimensional framework constructed from bi-octahedral Co_2_O_10_ dimers edge-connected to As_3_O_10_ groups. It delimits two sets of tunnels, running parallel to the *a* and *b* axes, the latter being the larger. The Li^+^ ions are located within the inter­sections of the tunnels. The possible motion of the alkali cations has been investigated by means of the BVS model. This simulation shows that the Li^+^ motion appears to be easier mainly along the *b*-axis direction and that this material may possess inter­esting conduction properties.

## Related literature

The investigated compound is the only arsenic member of the isotypic analogues Li*M*_2_ *X*_3_O_10_ (*M =* Fe, Co, Ni; *X =* P, As; Erragh *et al.*, 1996 ▶; Ramana *et al.*, 2006 ▶). For bond-valence-sum analysis, see: Brown (2002 ▶); Adams (2003 ▶). For the charge-distribution method, see: Nespolo (2001 ▶); Nespolo *et al.* (2001 ▶); Guesmi *et al.* (2006 ▶). For BVS pathway simulation, see: Mazza (2001 ▶); Ouerfelli *et al.* (2007 ▶). For a related compound, see: Satya Kishore & Varadaraju (2006 ▶).

## Experimental

### 

#### Crystal data

LiCo_2_As_3_O_10_
*M* *_r_* = 509.56
Monoclinic, 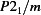

*a* = 4.830 (2) Å
*b* = 8.721 (2) Å
*c* = 9.3269 (9) Å
β = 98.08 (3)°
*V* = 388.97 (19) Å^3^
*Z* = 2
Mo *K*α radiation
μ = 16.97 mm^−1^
*T* = 298 K
0.14 × 0.10 × 0.07 mm

#### Data collection

Enraf–Nonius CAD-4 diffractometer
Absorption correction: ψ scan (North *et al.*, 1968 ▶) *T*_min_ = 0.200, *T*_max_ = 0.383
2027 measured reflections
903 independent reflections
816 reflections with *I* > 2σ(*I*)
*R*_int_ = 0.024
2 standard reflections every 120 reflections intensity decay: 3%

#### Refinement

*R*[*F* ^2^ > 2σ(*F* ^2^)] = 0.023
*wR*(*F* ^2^) = 0.063
*S* = 1.10
903 reflections
82 parameters
1 restraint
Δρ_max_ = 1.13 e Å^−3^
Δρ_min_ = −0.90 e Å^−3^

### 

Data collection: *CAD-4 EXPRESS* (Enraf–Nonius, 1995 ▶); cell refinement: *CAD-4 EXPRESS*; data reduction: *XCAD4* (Harms & Wocadlo, 1995 ▶); program(s) used to solve structure: *SHELXS97* (Sheldrick, 2008 ▶); program(s) used to refine structure: *SHELXL97* (Sheldrick, 2008 ▶); molecular graphics: *DIAMOND* (Brandenburg, 2001 ▶); software used to prepare material for publication: *WinGX* (Farrugia, 2012 ▶) and *publCIF* (Westrip, 2010 ▶).

## Supplementary Material

Crystal structure: contains datablock(s) I, global. DOI: 10.1107/S1600536813013548/br2224sup1.cif

Structure factors: contains datablock(s) I. DOI: 10.1107/S1600536813013548/br2224Isup2.hkl

Additional supplementary materials:  crystallographic information; 3D view; checkCIF report

## Figures and Tables

**Fig. 1.**
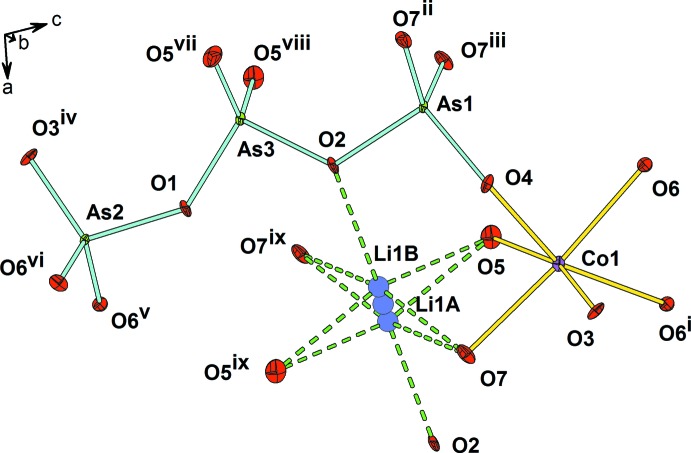
L'unité asymétrique dans LiCo2As3O10 (ellipsoïdes d'agitation thermique à 50% de probabilité; codes de symétrie: (i) -x+2, -y+2, -z+2; (ii) x-1, -y+3/2, z; (iii) x-1, y, z; (iv) x-1, y, z-1; (v) x, y, z-1; (vi) x, -y+3/2, z-1; (vii) -x+1, y-1/2, -z+1; (viii) -x+1, -y+2, -z+1;(ix) -x+2, -y+2, -z+1).

**Fig. 2.**
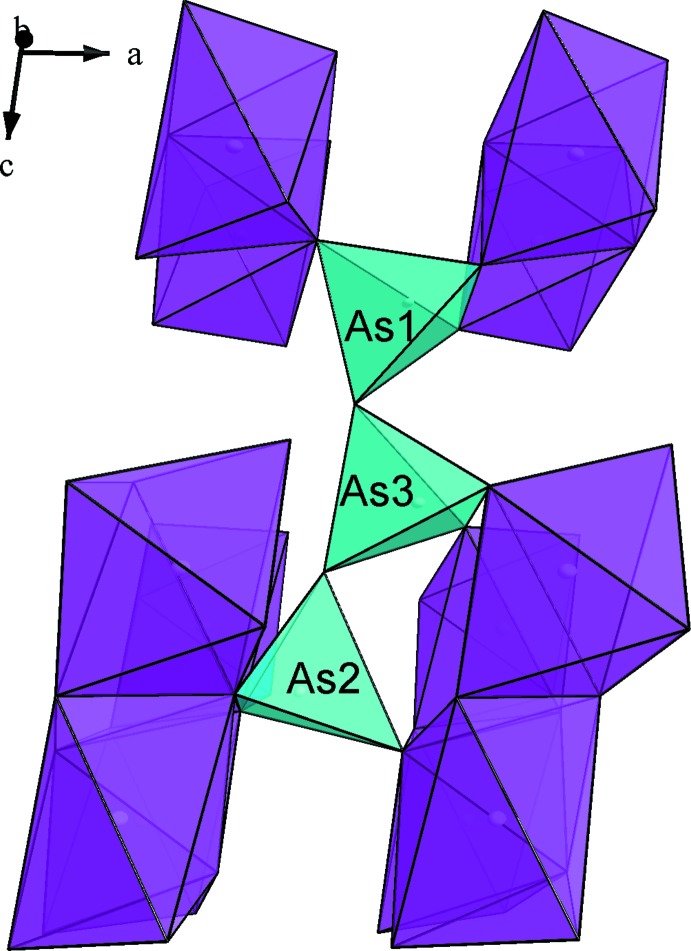
Modes de connexion entre les tétraèdres d'arsenic et les octaèdres de cobalt.

**Fig. 3.**
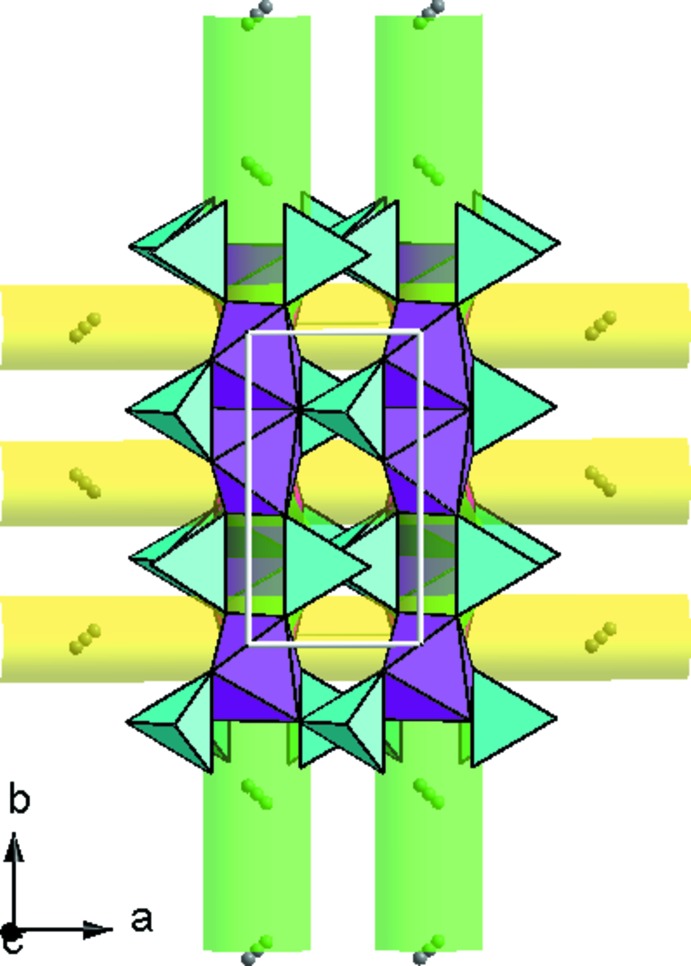
Vue en perspective montrant les deux types de tunnels matérialisés par des tubes.

**Fig. 4.**
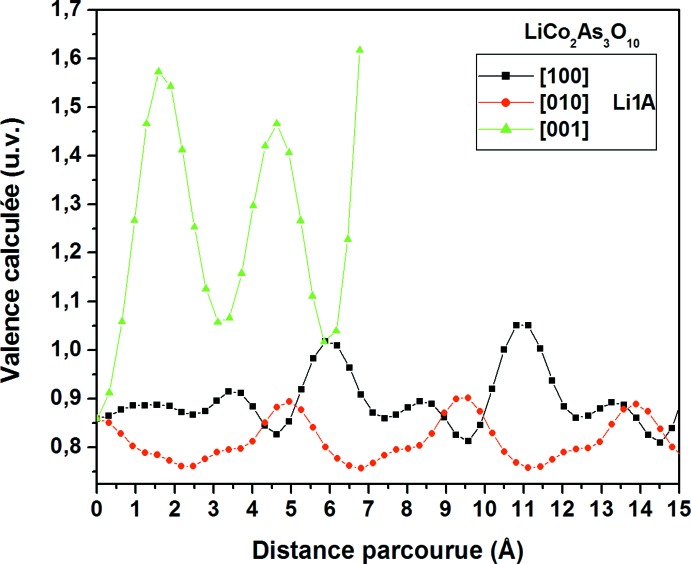
Analyse BVS de la mobilité des cations alcalins.

## supplementary crystallographic information

### Comment

L'exploration du système Li–Co–As–O se situe dans un contexte général visant la synthèse de nouveaux arséniates cristallisés, la détermination de leurs structures cristallines et l'étude de leurs propriétés électriques, à la recherche de nouveaux conducteurs au lithium pouvant constituer de nouveaux vecteurs d'énergie. Par ailleurs, un seul composé dans ce système, LiCoAsO_4_, est mentionné dans la littérature (Satya Kishore & Varadaraju, 2006).

Le matériau étudié de formulation LiCo_2_As_3_O_10_ a été synthétisé par réaction à l'état solide. Il est le seul arséniate de la famille des composés isotypes anhydres de formulation Li*M*_2_*X*_3_O_10_ (*M*: Fe, Co, Ni; *X*: P, As) (Erragh *et al.*, 1996; Ramana *et al.*, 2006). La structure est confirmée par les deux modèles de validation: le calcul des valences de liaisons BVS (Brown, 2002; Adams, 2003) et la méthode de distribution de charge CHARDI (Nespolo *et al.*, 2001; Nespolo, 2001) (Tableau 1).

L'unité asymétrique renferme un octaèdre de coordination du cobalt, trois tétraèdres formant le groupement As_3_O_10_ et un cation alcalin statistiquement désordonné sur deux positions (Fig. 1). Les différentes coordinences sont assurées par sept atomes d'oxygène cristallographiquement indépendants. Les valences calculées des différents atomes sont en bon accord avec les degrés d'oxydation. Quelques écarts sont observés pour un atome d'oxygène et comme dans des cas similaires pour les cations désordonnés. L'écart observé pour ces derniers est dû au fait que le désordre de position et donc l'occupation partielle se répercute sur les distances interatomiques et certaines seront légèrement supérieures à celles pour une occupation totale, et la valence diminue ainsi avec l'accroissement de ces distances.

L'analyse CHARDI confirme aussi le modèle structural proposé, notamment les charges des cations. Le facteur de dispersion sur ces charges est de 3% (Nespolo, 2001). Pour les charges anioniques, bien que le modèle CHARDI ne présente pas le même formalisme pour les cations et les anions (un écart par rapport aux valeurs idéales pour les anions est attribué à un effet OUB (*Over-Under Bonding effect*) (Nespolo *et al.* 2001), les charges anioniques sont confirmées avec un facteur de dispersion sur ces valeurs de 8%. L'effet OUB est observé pour les atomes d'oxygène tri-coordinés.

Le résultat de l'analyse CHARDI est fructueux et montre une autre vision des sphères de coordination par le biais du "nombre de coordination effectif ECoN". L'octaèdre de cobalt est assez distordu avec un nombre de coordination effectif de 5,94. Les distances moyenne classique d_moy_ et arithmétique pondérée d_med_ sont cependant très proches et sont respectivement de 2,114 e t 2,110 Å. Les distances interatomiques As—O varient globalement de 1,64 à 1,76 Å. Deux distances longues sont relevées pour les oxygènes des ponts As—O—As et la coordinence de l'entité centrale As(3)O_3_ du groupement As_3_O_10_ est ainsi du type 2 + 2. Le tétraèdre As(1)O_4_ avec une coordinence du type 3 + 1 présente la distance la plus longue. La distortion est ainsi plus prononcée pour ces deux tétraèdres avec des ECoNs de 3,92.

Les octaèdres partageant des arêtes sont arrangés en chaînes ondulées selon la direction [010], les distances Co—Co sont de 3,22 Å. Les groupements As_3_O_10_ situés à *y* = ±1/4 relient ces chaînes par partage de sommets avec les octaèdres. Chaque groupement met en commun ses huit sommets avec dix octaèdres de quatre chaînes parallèles (Fig. 2) et quelques atomes d'oxygène sont donc tri-coordinés.

La charpente anionique résultante montre de larges tunnels selon la direction *b* et d'autres moins larges selon la direction *a* (Fig. 3). Les cations alcalins statistiquement désordonnés occupent ces tunnels et apparaissent à l'intersection de leurs sections. Les cations Li1A occupent un centre d'inversion (position *2d*) avec une coordination ayant la forme d'un plan carré; ces mêmes caractéristiques sont signalées dans les phosphates isotypes. La différence vient cependant de l'existence du désordre et donc de la deuxième position générale avec des distances Li—O allant jusqu'à 2,64 Å. Cet "éclatement" est attribué à l'augmentation des dimensions des tunnels et un cation petit comme le lithium tente à gagner la périphérie du tunnel ou de la cavité occupée pour compléter sa coordinence.

Pour avoir une idée préliminaire sur la mobilité des cations alcalins, le modèle BVS semble être, au delà de la validation, un moyen fructueux. Ce concept est détaillé dans plusieurs travaux antérieurs (Mazza, 2001; Ouerfelli *et al.* 2007). L'analyse par le concept BVS de la structure de l'arséniate étudié montre que les directions [100] et [010] sont favorables pour la mobilité du lithium où la somme de valences est proche de la valence idéale (Fig. 4). En effet, la valence maximale Vmax selon la direction initiale *b* est au voisinage de 0,91 u.v. pour des distances de migration qui dépassent 10 Å. Les valences calculées pour la direction [100] dépassent légèrement la valence idéale (Vmax = 1,05 u.v.), cette direction est donc moins favorable que la première. Pour les autres directions, les valences calculées sont élevées (par exemple: Vmax > 1,7 u.v. pour [001]), le cation mobile rencontre donc des barrières énergétiques exercées par la charpente anionique. La modélisation BVS montre ainsi que cette classe de matériaux peut constituer des matrices importantes pour des propriétés électriques. Ceci n'exclut pas une étude expérimentale des ces propriétés, ce travail est actuellement en cours.

### Experimental

Le nouvel arséniate étudié a été synthétisé par réaction à l'état solide. Un mélange de Li_2_CO_3_ (AZIENDA CHIMICA. 99,5%), CoCl_2_.6H_2_O (PARK. 99%) et de NH_4_H_2_AsO_4_ (préparé au laboratoire, ASTM 01–775), pris dans les proportions molaires Li:Co:As =1:1:3 a été finement broyé dans un mortier en agate. Il a été mis dans une nacelle en porcelaine et calciné à 623 K pendant 12 heures. Après refroidissement, le mélange a été broyé de nouveau puis porté à 1003 K et maintenu à cette température pendant 3 jours. Il a été ensuite refroidi lentement avec une vitesse de 5 K/h jusqu'à 953 K puis finalement refroidi jusqu'à la température ambiante. Des cristaux de couleur rose sont apparus sur les parois de la nacelle. Cette dernière a été lavée à l'eau bouillante afin de séparer les cristaux du flux. Les cristaux ont été ensuite triés sous une loupe binoculaire et l'échantillon sélectionné pour la collecte des données a été choisi après examen sous un microscope à lumière polarisée.

### Refinement

L'affinement de l'un des atomes d'oxygène (O2) des ponts As—O—As n'était pas possible en anisotropie. Il a été contraint à avoir les mêmes facteurs d'agitation thermique anisotrope que l'atome O1 (option EADP du programme SHELX) des mêmes ponts. Pour les cations alcalins, la somme des taux d'occupation des deux sites correspond à la valeur qui assure la neutralité électrique.

### Crystal data

**Table:**

LiCo_2_(As_3_O_10_)	*F*(000) = 472
*M**_r_* = 509.56	*D*_x_ = 4.351 Mg m^−^^3^
Monoclinic, *P*2_1_/*m*	Mo *K*α radiation, λ = 0.71073 Å
Hall symbol: -P 2yb	Cell parameters from 25 reflections
*a* = 4.830 (2) Å	θ = 12.4–14.8°
*b* = 8.721 (2) Å	µ = 16.97 mm^−^^1^
*c* = 9.3269 (9) Å	*T* = 298 K
β = 98.08 (3)°	Parallelepiped, pink
*V* = 388.97 (19) Å^3^	0.14 × 0.10 × 0.07 mm
*Z* = 2	

### Data collection

**Table:**

Enraf–Nonius CAD-4 diffractometer	816 reflections with *I* > 2σ(*I*)
Radiation source: fine-focus sealed tube	*R*_int_ = 0.024
Graphite monochromator	θ_max_ = 27.0°, θ_min_ = 2.2°
ω/2θ scans	*h* = −6→6
Absorption correction: ψ scan (North *et al.*, 1968)	*k* = −1→11
*T*_min_ = 0.200, *T*_max_ = 0.383	*l* = −11→11
2027 measured reflections	2 standard reflections every 120 reflections
903 independent reflections	intensity decay: 3%

### Refinement

**Table:**

Refinement on *F*^2^	Primary atom site location: structure-invariant direct methods
Least-squares matrix: full	Secondary atom site location: difference Fourier map
*R*[*F*^2^ > 2σ(*F*^2^)] = 0.023	*w* = 1/[σ^2^(*F*_o_^2^) + (0.0372*P*)^2^ + 0.4094*P*] where *P* = (*F*_o_^2^ + 2*F*_c_^2^)/3
*wR*(*F*^2^) = 0.063	(Δ/σ)_max_ = 0.007
*S* = 1.10	Δρ_max_ = 1.13 e Å^−^^3^
903 reflections	Δρ_min_ = −0.90 e Å^−^^3^
82 parameters	Extinction correction: *SHELXL97* (Sheldrick, 2008), Fc^*^=kFc[1+0.001xFc^2^λ^3^/sin(2θ)]^-1/4^
1 restraint	Extinction coefficient: 0.0240 (13)

### Special details

**Table:**

Geometry. All esds (except the esd in the dihedral angle between two l.s. planes) are estimated using the full covariance matrix. The cell esds are taken into account individually in the estimation of esds in distances, angles and torsion angles; correlations between esds in cell parameters are only used when they are defined by crystal symmetry. An approximate (isotropic) treatment of cell esds is used for estimating esds involving l.s. planes.
Refinement. Refinement of F^2^ against ALL reflections. The weighted R-factor wR and goodness of fit S are based on F^2^, conventional R-factors R are based on F, with F set to zero for negative F^2^. The threshold expression of F^2^ > 2sigma(F^2^) is used only for calculating R-factors(gt) etc. and is not relevant to the choice of reflections for refinement. R-factors based on F^2^ are statistically about twice as large as those based on F, and R- factors based on ALL data will be even larger.

### Fractional atomic coordinates and isotropic or equivalent isotropic displacement parameters (Å^2^)

**Table:**

	*x*	*y*	*z*	*U*_iso_*/*U*_eq_	Occ. (<1)
Co1	1.03666 (8)	0.93447 (6)	0.83555 (4)	0.00531 (17)	
As1	0.45631 (9)	0.7500	0.65408 (5)	0.00377 (16)	
As2	0.61125 (9)	0.7500	0.02638 (5)	0.00327 (16)	
As3	0.33999 (10)	0.7500	0.32284 (5)	0.00419 (16)	
O1	0.5921 (7)	0.7500	0.2116 (4)	0.0104 (5)	
O2	0.5743 (7)	0.7500	0.4830 (4)	0.0104 (5)	
O3	1.2865 (7)	0.7500	0.9368 (4)	0.0070 (7)	
O4	0.7674 (7)	0.7500	0.7587 (4)	0.0070 (7)	
O5	0.8393 (5)	1.0887 (3)	0.6836 (3)	0.0116 (5)	
O6	0.7861 (5)	0.9132 (3)	1.0088 (3)	0.0071 (5)	
O7	1.2675 (5)	0.9089 (3)	0.6622 (3)	0.0086 (5)	
Li1A	1.0000	1.0000	0.5000	0.007 (3)*	0.54 (7)
Li1B	0.947 (8)	0.973 (4)	0.500 (3)	0.007 (3)*	0.23 (4)

### Atomic displacement parameters (Å^2^)

**Table:**

	*U*^11^	*U*^22^	*U*^33^	*U*^12^	*U*^13^	*U*^23^
Co1	0.0062 (3)	0.0054 (3)	0.0042 (3)	−0.00006 (14)	0.00014 (19)	−0.00079 (15)
As1	0.0035 (2)	0.0057 (3)	0.0019 (3)	0.000	−0.00053 (18)	0.000
As2	0.0028 (2)	0.0041 (3)	0.0026 (3)	0.000	−0.00044 (18)	0.000
As3	0.0043 (2)	0.0056 (3)	0.0024 (3)	0.000	−0.00020 (17)	0.000
O1	0.0079 (10)	0.0216 (13)	0.0021 (11)	0.000	0.0021 (9)	0.000
O2	0.0079 (10)	0.0216 (13)	0.0021 (11)	0.000	0.0021 (9)	0.000
O3	0.0048 (15)	0.0064 (15)	0.0083 (17)	0.000	−0.0039 (13)	0.000
O4	0.0074 (15)	0.0079 (15)	0.0045 (15)	0.000	−0.0029 (13)	0.000
O5	0.0129 (12)	0.0103 (11)	0.0111 (12)	0.0059 (10)	−0.0006 (10)	0.0012 (10)
O6	0.0071 (11)	0.0070 (10)	0.0076 (12)	−0.0045 (10)	0.0021 (9)	−0.0032 (10)
O7	0.0104 (11)	0.0088 (11)	0.0076 (11)	0.0050 (10)	0.0047 (9)	0.0020 (10)

### Geometric parameters (Å, º)

**Table:**

Co1—O6^i^	2.063 (3)	As3—O5^viii^	1.649 (3)
Co1—O5	2.085 (3)	As3—O1	1.707 (3)
Co1—O7	2.100 (3)	As3—O2	1.743 (4)
Co1—O4	2.129 (2)	Li1A—Li1B	0.35 (5)
Co1—O3	2.148 (2)	Li1A—O7	2.010 (2)
Co1—O6	2.159 (2)	Li1A—O7^ix^	2.010 (2)
As1—O7^ii^	1.667 (2)	Li1A—O5	2.123 (3)
As1—O7^iii^	1.667 (2)	Li1A—O5^ix^	2.123 (3)
As1—O4	1.673 (3)	Li1B—Li1B^ix^	0.70 (10)
As1—O2	1.768 (3)	Li1B—O7^ix^	1.99 (3)
As2—O3^iv^	1.670 (3)	Li1B—O7	2.08 (3)
As2—O6^v^	1.675 (2)	Li1B—O5	2.11 (3)
As2—O6^vi^	1.675 (2)	Li1B—O5^ix^	2.19 (3)
As2—O1	1.743 (3)	Li1B—O2	2.64 (3)
As3—O5^vii^	1.649 (3)		
			
O6^i^—Co1—O5	99.67 (12)	O7^iii^—As1—O4	115.20 (10)
O6^i^—Co1—O7	113.41 (10)	O7^ii^—As1—O2	106.80 (10)
O5—Co1—O7	77.67 (10)	O7^iii^—As1—O2	106.80 (10)
O6^i^—Co1—O4	153.78 (12)	O4—As1—O2	98.59 (16)
O5—Co1—O4	93.40 (11)	O3^iv^—As2—O6^v^	113.71 (10)
O7—Co1—O4	91.49 (12)	O3^iv^—As2—O6^vi^	113.71 (10)
O6^i^—Co1—O3	91.20 (10)	O6^v^—As2—O6^vi^	116.39 (17)
O5—Co1—O3	163.09 (12)	O3^iv^—As2—O1	108.59 (17)
O7—Co1—O3	86.15 (12)	O6^v^—As2—O1	101.23 (10)
O4—Co1—O3	82.01 (11)	O6^vi^—As2—O1	101.23 (10)
O6^i^—Co1—O6	75.48 (10)	O5^vii^—As3—O5^viii^	117.15 (18)
O5—Co1—O6	108.27 (10)	O5^vii^—As3—O1	113.20 (10)
O7—Co1—O6	168.82 (11)	O5^viii^—As3—O1	113.20 (10)
O4—Co1—O6	78.87 (11)	O5^vii^—As3—O2	107.79 (11)
O3—Co1—O6	86.90 (12)	O5^viii^—As3—O2	107.79 (11)
O7^ii^—As1—O7^iii^	112.51 (17)	O1—As3—O2	95.06 (17)
O7^ii^—As1—O4	115.20 (10)		

Symmetry codes: (i) −*x*+2, −*y*+2, −*z*+2; (ii) *x*−1, −*y*+3/2, *z*; (iii) *x*−1, *y*, *z*; (iv) *x*−1, *y*, *z*−1; (v) *x*, *y*, *z*−1; (vi) *x*, −*y*+3/2, *z*−1; (vii) −*x*+1, *y*−1/2, −*z*+1; (viii) −*x*+1, −*y*+2, −*z*+1; (ix) −*x*+2, −*y*+2, −*z*+1.

### Analyses CHARDI et BVS relatives aux cations dans LiCo_2_As_3_O_10_.

**Table:**

Cation	q(i).sof(i)	Q(i)	V(i)	CN(i)	ECoN(i)	d_moy_	d_med_
As1	5,00	5,043	4,987	4	3,915	1,693	1,687
As2	5,00	4,967	5,039	4	3,958	1,690	1,687
As3	5,00	4,958	5,007	4	3,921	1,686	1,681
Co1	2,00	2,015	2,014	6	5,944	2,114	2,110
Li1*A*	0,54	0,540	0,464	4	3,894	2,067	2,056
Li1*B*	0,23	0,230	0,194	5	3,951	2,205	2,085

q(i)= nombre d'oxydation, sof(i)= taux d'occupation du site, Q(i)= charge calculée, CN= nombre de coordination classique, ECoN= nombre de coordination effectif, σ=[Σi(q_i_-Q_i_)^2^/N-1]^1/2^=0,032.
